# Innovation, Automation and Informatics Improves Quality in Lerdsin Hospital, Thailand

**DOI:** 10.3389/bjbs.2023.11532

**Published:** 2023-06-19

**Authors:** Adchada Karnchanaphiboonwong, Patcharawadee Sringam, Kawinna Niwattakul, Teerayut Krommuang, Alistair Gammie

**Affiliations:** ^1^ Central Laboratory, Lerdsin Hospital, Bangkok, Thailand; ^2^ Quidel Ortho Diagnostics, Pencoed, United Kingdom

**Keywords:** informatics, innovation, TAT, turnaround time, automation, Vitros automation system

## Abstract

This paper describes a planned, continuous improvement journey, of a laboratory that has installed a system with a single sample touch from blood draw to result. To achieve this, physical connectivity of systems from phlebotomy through pre-analytical to the analytical phase were paired with informatics connectivity from the patient’s national identity card to the hospital and laboratory informatics management systems (LIMS) and associated middleware. This allowed accurate time stamps to track turnaround time (TAT). TAT metrics were collected from the LIMS for inpatient, emergency room and outpatient samples and tests over a period of 7 months. This time span incorporated the 2-month period before automation was implemented. The results for all tests and specific tests are shown and the results of an analysis of the outpatient phlebotomy workflow are given. The implemented solution has improved outpatient TAT by over 54% and has shown that samples can be collected, and results obtained without touching the sample. Improving intra-laboratory TAT is an important quality goal for all laboratories. The implementation of automation is important in achieving this albeit more about obtaining predictable TAT. Automation does not necessarily improve TAT it removes variation which leads to predictable TAT (PTAT). Automation should only be considered with a strategic vision for the future as it is important to have clear goals and objectives based on the individual laboratories process and needs. Automating a poor process leads to an automated poor process. Here, an innovative use of automation, hardware and software has resulted in marked improvement in TAT across all samples processed in the central laboratory.

## Introduction

Lerdsin Hospital is a 737-bed tertiary care hospital in Bangrak district of Bangkok with a Medical School founded in 1889 and was the first private hospital in Thailand. The hospital became a government hospital after World War 2.

The laboratory provides multidisciplinary service for both in and outpatients. In 2022, a Quidel Ortho Vitros automation system (VAS) (Ortho Clinical Diagnostics, New Jersey, US) was installed with an aspirational objective that blood samples should be touched only once.

Prolonged turnaround times (TAT) are recognized as posing a significant risk to patient safety, and there is some evidence they also impact clinician and patient satisfaction as well as increased length of stay. Although the principles of target and maximum standards have been adopted by other specialties there are no agreed standards for TAT in the laboratory. Auld et al describe the study run by the National Clinical Biochemistry audit group (UK) in 2016 looking at minimum and maximum TAT limits for the Emergency Room (ER), in-patient (IPD), outpatient (OPD) and General Practice (GP). There was good consensus around the optimum TAT target for ER and IPD (<1 h and 4 h) although there was less agreement about OPD and GP with a split between <12 h or <24 h [1]. Dawande et al state that TAT should be broadly divided into three stages preanalytical, analytical and post-analytical and describe many of the reasons that can affect TAT in each phase. These include how phlebotomy is organised, choice of analytical equipment and maintenance as well as how reports are made available to clinicians [2]. Khalifa et al also stress that timeliness is an essential laboratory quality indicator, but they put the focus more on the pre-analytical process as the predominant issue and consider the time taken to get samples from ER to the laboratory and the use of point of care testing (POC) [3]. Stotler et al believe that lack of full control over phlebotomy and specimen transport make it difficult for the laboratory to address delays caused in the preanalytical stages that take place outside the laboratory. In addition, ordering and collection times are not always fully documented for all samples, and it is currently not possible to determine when clinicians become aware of most laboratory results. Thus, it is impossible for laboratories to use the total TAT as a quality assurance measure. They studied intra laboratory TAT and identified that specimen debagging and accessioning was an issue in their process and added two staff to that area and showed a significant improvement in the TAT [4]. Improving turnaround time is rarely a matter of implementing one-size-fits-all solutions; it requires participation from the entire team and implementing strategies that make sense within the lab’s framework. These strategies included Lean 6 Sigma, installing middleware and automation, auto-verification, centralised areas for equipment, reduce time between sample arrival and accessioning, and reliable barcode labelling [5].

Intra-laboratory TAT is an acceptable measure as it can be accurately measured especially if electronic patient requesting is used and samples are auto-receipted in the automation system. The measurements were made in line with those of Angeletti et al [6]. In this study, the improvement in intra-laboratory TAT for IPD was measured by calculating the time of arrival at the laboratory from the pneumatic tube system to the release of result. The OPD TAT was measured from patient arrival in the phlebotomy area to release of results as the complete workflow was optimized and allowed us to have an accurate TAT from patient arrival to release of results.

The standalone analysers were installed in March 2022, and the phlebotomy department was moved in April 2022. The automation system was introduced in June 2022. The automation system automatically registers the patient on arrival. The samples are then centrifuged, decapped, aspirated or sorted for offline testing as required. Haematology, coagulation, and flow cytometry are not directly connected to the automation, but the tubes are delivered to the appropriate area of the laboratory.

The aspirational goal of the laboratory was to deliver all chemistry results in 1 h and all immunoassay within 2 h. Comparison of the data using the same analytical solution before and after automation and post implementation of the new OPD process allowed us to measure the effects of the new processes. In addition, after the preliminary results were calculated a review of the OPD phlebotomy workflow using available timestamps was made to calculate the impact of this portion of the workflow.

## Materials and Methods

The laboratory information and management system (LIMS) is HCLAB, Singapore (HC Lab Sysmex AP, Singapore); all requests to the laboratory are made electronically. The samples arrive from ward areas by air tube together with its associated order form containing a bar code, which is scanned. The tube bar codes are printed and affixed, and the sample is introduced either into the bulk input module or the ES Flex entry module (Quidel Ortho Diagnostics, New Jersey, US). In the current study, a ValuMetrix™ Lean based consulting exercise had been performed to document and streamline the processes, an automation system with associated middleware had been installed with auto-verification planned in the next phase. The core lab was designed so that sample delivery was integrated or within 2 m of the delivery point for >95% of samples. In addition, the out-patient phlebotomy process was connected to the automation system and used auto-labellers to standardise the tube labelling process.

It had been identified in the consultation that OPD bloods were drawn on the first floor utilizing a sample administration and queuing system provided by Bangkok Inter Products (Bangkok, Thailand). There were also auto-labelling stations that were in use 50% of the time when samples were transported to the laboratory area by a sample lift or by foot. Analysis showed that this increased variation of the TAT. However, this data is not included in this paper, as it cannot be directly compared to the current data points. When the new laboratory layout was designed, the administration and queuing system for phlebotomy were moved to the same floor as the laboratory. The auto-labellers were then put into 100% use to achieve correct draw order and correctly positioned barcode labels to enable error free labelling of the tubes. The six phlebotomy stations were connected by a conveyor (CV Design, Samutprakarn, Thailand) which moved the samples directly into the laboratory. This has a direct connection with a bulk entry module so that samples can automatically register the patient identity on arrival (in-labbing) using cap colour detection to assure that the correct tube is being used.

Outpatients arriving for phlebotomy report to sample administration with their request form, which has a bar code associated with their order. If the form has no bar code the patient can use either the bar code or chip of their Thailand identity card. The patient is given their queue number and QR code then sits down watching the screens for their number to be displayed and which cubicle to go to. The QR code generates the auto labelling system to print the barcodes and attach them to the correct tubes in the correct draw order.

Once the blood is drawn, all tubes are placed onto the conveyor system, which connects each of the phlebotomy stations and then enters the laboratory through a port in the wall.

**FIGURE 1:**
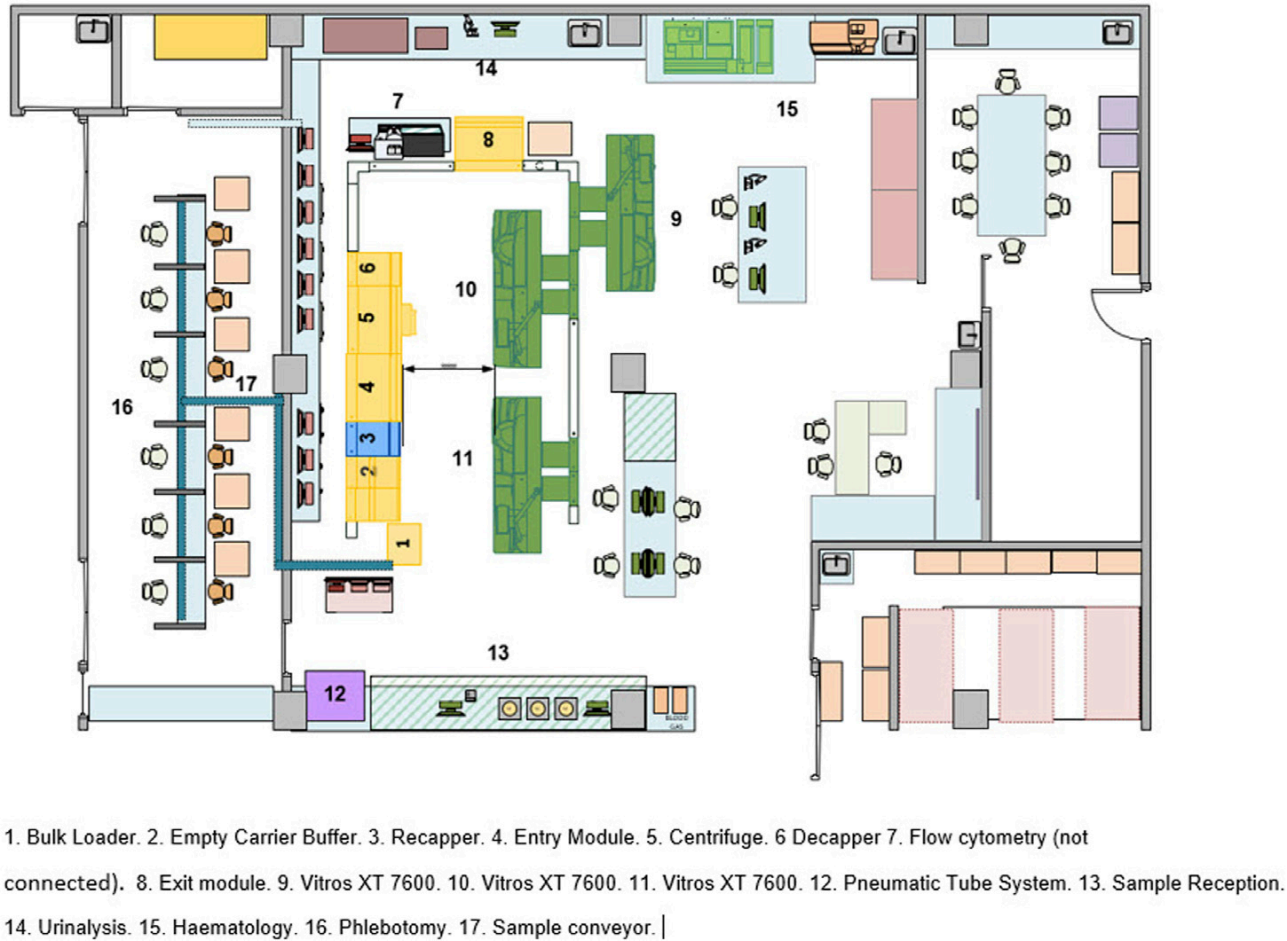
Vitros automation system layout.

The automation system is placed directly behind the phlebotomy stations separated by a wall with large picture windows that allows patients to see how their samples are processed. The automation system is a Vitros Automation System (VAS) comprising a bulk entry module (bulk input module and sample transport and identification module), two ES Flex input output modules, centrifuge, decapper, 3 Vitros XT 7600™ integrated chemistry analyzers, and a recapper shown in Figure 1.

The tubes travel from the bulk entry module where they are registered onto the LIMS and the tubes are routed to the centrifuge, decapper, analyser or ES Flex exit (#8) for sorting as required by the test ordered. Post testing samples are sent back to the ES Flex (#8) exit module where they are registered for storage or sorted for further offline testing on the ES Flex (#4). IPD and ER blood samples arrive through the pneumatic tube system are scanned to register and either placed onto the ES Flex (#4) if they are urgent or placed into the bulk entry module. Post testing, they follow the same route as the OPD samples.

The first phase of this project reported in this paper was the introduction of stand-alone Vitros XT7600™ analyzers that were installed in March 2022 with the new OPD workflow going live at the end of May but phlebotomy was not connected to the laboratory systems at this time. The second phase was the introduction of the automation system in June 2022 which linked up the informatic workflow of the phlebotomy process with the pre–analytical process and the smart routing principles of Instrument Manager Solution for Vitros Automation (IMSVA) (Quidel Ortho Diagnostics, New Jersey, US) which not only performs load balancing, reroutes samples when there are reagent outages but also has the Instructions for Use (IFU) for all Vitros tests embedded within the software. This allows automatic repeats and dilutions where appropriate and retains the one touch objective. The third phase was auto-verification that was due to be introduced in December 2022, but this has been delayed due to the introduction of a new hospital information system. All data collected for TAT analysis was collected from the LIS or the phlebotomy management system.

In addition to looking at the TAT data for all assays, it was also considered beneficial to look at individual assays to identify potential outliers and investigate the reasons. The following assays were chosen as being representative for STAT (high priority) and routine priorities and across disease states. Creatinine, random glucose, sodium, cholesterol, HIV combo (antigen/antibody assay), high-sensitivity troponin I and thyroid stimulating hormone (TSH). The mean TAT was calculated for each month and no outliers were removed. The difference between the means of the TAT for each individual assay across all three groups between the standalone analytics and fully automated periods were analysed using the Student’s t-test. This was done using the data analysis function in Microsoft Excel using the *t*-test ‘Paired two sample for means’ function.

## Results

### Overall TAT Reduction

The mean TAT for all tests including chemistry, haematology, immunology and coagulation from all locations in the hospital is shown in Table 1. There was some disruption in May whilst the automation system was being installed. However, since the highest TAT recorded during the implementation, there has been continual month on month improvement realizing a 47.6% decrease in TAT. Prior to the introduction of the new processes the mean TAT was over 2.5 h, and this decreased to below 1.5 h.

**TABLE 1:** Mean TAT (minutes) from all Locations for all tests.

All tests and locations	April	May	June	July	August	September	October
Grand Total (minutes)	154.68	168.45	124.85	104.18	95.45	89.59	88.26

### TAT by Department

A breakdown of TAT of individual tests from the ER, IPD and OPD are shown in Tables 2–4. ER and IPD TAT were measured from the time the form was scanned in the laboratory and OPD TAT was measured from the time the patient arrived at the administration desk. Every sample was processed through the automation system with online centrifugation and decapping where appropriate. STAT tests were loaded at the ES Flex and given priority at the analyzer bypasses. There has been continuous improvement in TAT from when the automation went live in June.

**TABLE 2:** Mean turnaround time by month for selected emergency room tests.

Test and location	April	May	June	July	August	September	October
Emergency Room	**89.25**	**76.51**	**71.36**	**77.17**	**66.60**	**69.89**	**64.78**
Anti-HIV	109.40	108.51	95.07	116.82	95.78	100.04	88.29
BUN	90.86	73.43	69.71	70.55	64.24	67.01	62.26
Calcium	92.78	75.51	73.86	78.56	66.95	69.08	65.58
Chloride	82.10	72.35	67.58	71.08	62.24	65.87	61.51
Cholesterol	202.39	89.55	93.11	126.69	86.00	111.87	93.55
CO2	82.72	72.50	68.33	71.72	63.06	66.41	62.28
Creatinine	83.47	73.48	69.30	72.15	63.50	66.60	62.17
Electrolyte	82.78	72.50	68.33	71.72	63.06	66.41	62.28
Glucose	81.09	76.22	71.41	66.04	70.27	71.66	61.86
LDL	200.75	90.27	93.11	127.12	86.00	111.87	93.26
Lipid Profile	203.14	90.61	93.11	127.12	86.00	111.87	93.26
Potassium	81.99	72.31	67.58	71.03	62.20	65.68	61.47
Sodium	81.97	72.30	67.57	70.94	62.20	65.71	61.49
Troponin I	70.47	72.87	64.15	58.72	63.88	60.49	56.70
TSH	88.52	120.66	109.78	77.25	75.77	84.02	72.37

Using the Student’s t-test “Paired two samples for means” the emergency room difference in TAT between April to June and July to October was significant at *p* < 0.05, the P(T ≤ t) two- tail result was 2.73^-5^.

The figures in bold are the average of all the tests in that patient segment.

**TABLE 3:** Mean turnaround time by month for selected inpatients tests.

Test and location	April	May	June	July	August	September	October
Inpatient Department	**81.95**	**79.99**	**73.43**	**68.08**	**68.58**	**66.64**	**62.72**
Anti-HIV	130.16	146.29	116.52	101.33	115.06	111.16	102.45
BUN	81.59	79.24	72.76	68.13	68.80	65.56	62.94
Calcium	83.72	85.01	69.18	65.17	62.68	67.19	59.63
Chloride	76.77	74.44	70.05	65.69	65.63	63.51	60.28
Cholesterol	111.73	91.05	104.37	76.89	91.59	92.47	75.74
CO_2_	76.88	74.51	70.16	66.08	66.01	63.65	60.47
Creatinine	81.64	79.19	72.64	67.85	67.76	65.34	62.14
Electrolyte	76.88	74.51	70.19	66.08	66.01	63.65	60.47
Glucose	91.45	102.02	74.75	68.55	66.31	62.97	63.91
LDL	110.14	95.62	103.17	77.09	93.59	90.65	76.58
Lipid Profile	104.34	91.05	102.73	77.36	91.59	91.55	75.74
Potassium	76.65	74.79	69.97	65.54	65.55	63.28	60.17
Sodium	76.58	74.46	70.07	65.47	65.52	63.47	60.19
Troponin I	104.45	84.75	82.45	65.34	59.55	81.60	59.98
TSH	177.96	149.62	127.44	102.52	95.40	96.99	83.81

Using the Student’s t-test “Paired two samples for means” the Inpatients Department difference in TAT between April to June and July to October was significant at *p* < 0.05, the P(T ≤ t) two-tail result was 2.03^-4^.

The figures in bold are the average of all the tests in that patient segment.

**TABLE 4:** Mean Turnaround time by month for selected outpatients tests.

Test and location	April	May	June	July	August	September	October
Outpatient Department	**214.24**	**232.20**	**165.19**	**132.78**	**116.94**	**106.26**	**107.75**
Anti-HIV	233.48	251.37	176.36	149.16	148.64	136.63	133.82
BUN	196.93	213.03	151.37	121.29	105.55	97.35	97.67
Calcium	217.92	238.54	157.29	127.88	115.27	105.02	104.51
Chloride	204.46	222.63	161.33	125.55	110.97	99.53	102.25
Cholesterol	227.69	245.16	171.92	141.98	127.37	113.19	115.15
CO_2_	204.67	222.84	161.48	125.55	111.05	99.61	102.21
Creatinine	213.23	228.34	163.52	133.43	116.77	107.24	109.83
Electrolyte	204.69	222.96	161.48	125.51	111.04	99.61	102.19
Glucose	221.01	242.60	175.11	141.84	122.36	111.64	113.63
LDL	224.57	243.29	169.62	135.25	115.05	106.73	108.02
Lipid Profile	225.28	244.96	170.58	135.73	116.46	106.07	106.88
Potassium	204.44	222.85	161.56	125.74	110.71	99.51	102.24
Sodium	204.64	222.95	161.22	125.61	111.29	99.60	102.32
Troponin I	154.13	155.37	121.69	104.47	104.90	92.06	83.78
TSH	239.17	251.62	165.93	144.48	124.19	114.22	113.27

Using the Student’s t-test “Paired two samples for means” the Outpatients Department difference in TAT between April to June and July to October was significant at *p* < 0.05, the P(T ≤ t) two-tail result was 1.25^-13^.

The figures in bold are the average of all the tests in that patient segment.

Although the majority of ER samples are high priority, the mean TAT dropped from a high of 83.02 to 61.76 min. Blood urea nitrogen reduced from 90.86 to 62.26 min and Troponin I from 72.87 to 56.70 min as can be seen in Table 2.

IPD test (excluding ER) mean TAT dropped from 81.59 to 62.94 min, see Table 3. Blood urea nitrogen reduced from 90.86 to 62.26 min and Troponin I from 104.45 to 59.98 min. HIV antibody testing reduced from a high of 146.29 to 102.45 min although month on month reductions were not as marked as in other markers. This is attributable in some part to the fact that positive results require retesting before a result can be posted.

OPD test mean TAT dropped from 214.24 to 107.75 min, see Table 4. Blood urea nitrogen reduced from 196.93 to 97.67 min and Troponin I from 154.13 to 83.78 min.

IPD TAT decreased by 23.44%, ER TAT by 27.41% and OPD TAT by 54.24%; for ER and IPD the aspirational goal of 60 min for chemistry and 120 min for immunoassay are close to achievement and the move to auto-verification should result in this goal being met.

### Monthly Test Volumes

**FIGURE 2:**
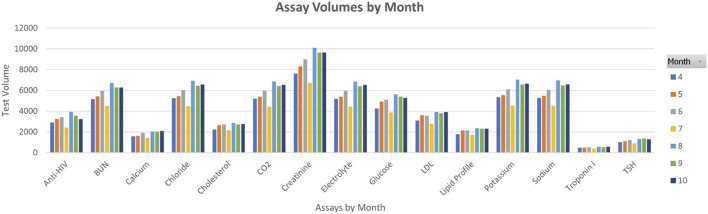
Monthly test volumes.

The split between locations remains stable at 14% ER, 32% IPD and 54% OPD and the monthly test volumes are shown in Figure 2. The ER test volume has increased from 21,985 to 28,612. The last 3 months have seen an increase in testing volume for all tests except for troponin I. The volume of work for the three departments was measured from April to June and compared with that of August to October. The workload increased by 30% in ER, 14% in IPD, and a 18% increase in OPD in the latter period, as shown in Table 5, even as the TAT improved.

**TABLE 5:** Workload increase between April and October.

	Percentage workload increase		
	Emergency Room (ER)	Inpatient Department (IPD)	Outpatient Department (OPD)
April-June	21,985	55,265	99,858
August–October	28,612	63,003	117,876
Percentage increase	30%	14%	18%

### OPD Phlebotomy TAT

**FIGURE 3:**
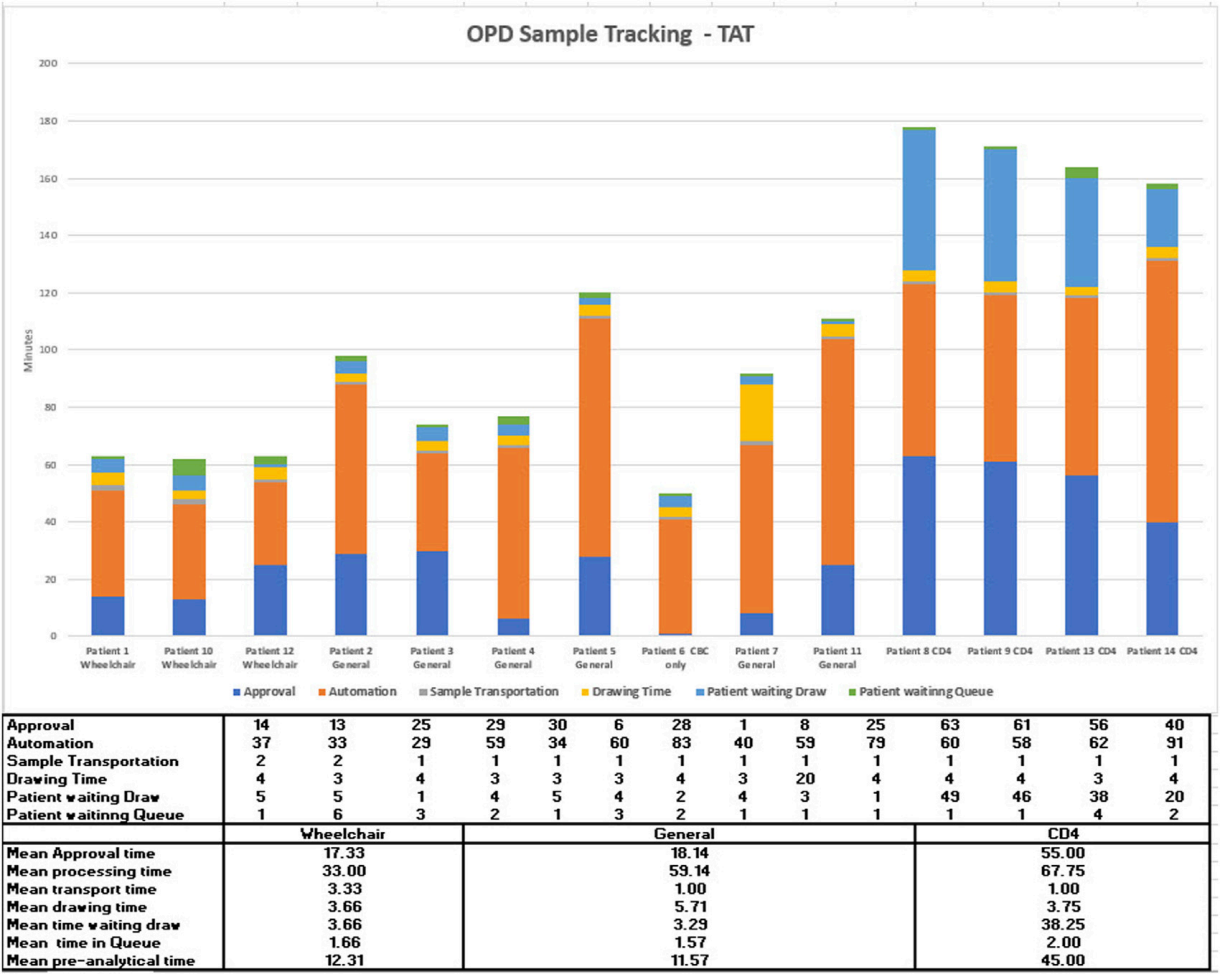
Outpatient department phlebotomy workflow study.

After the first 3 months of comparative data was collected, a study of the phlebotomy workflow was made following individual patients from sample administration through to result validation using available time stamps. These results are shown in Figure 3, the patients are numbered in their order of arrival but are shown by patient type, General are patients who go through the routine process, wheelchair patients are bled in their chairs and CD4 patients require a specialist nurse. For patients waiting to get their queue numbers the mean wait time was 2.07 min, the median 1.5 min, and the maximum 6 min. The wait time for phlebotomy was compromised by 4 patients who turned up earlier than their appointment time and there was no specialist nurse available to bleed them (patients 8, 9, 13, and 14). This waiting time is included within the overall TAT data. The mean wait time for rest of the patients was 3.5 min and the median was 4 min. The mean and median blood collection times were 4.7 and 4 min, respectively. On average, a patient stays for 10.17 min, from entry into the OPD phlebotomy area. This sets an aggressive target for continuous improvement.

**FIGURE 4:**
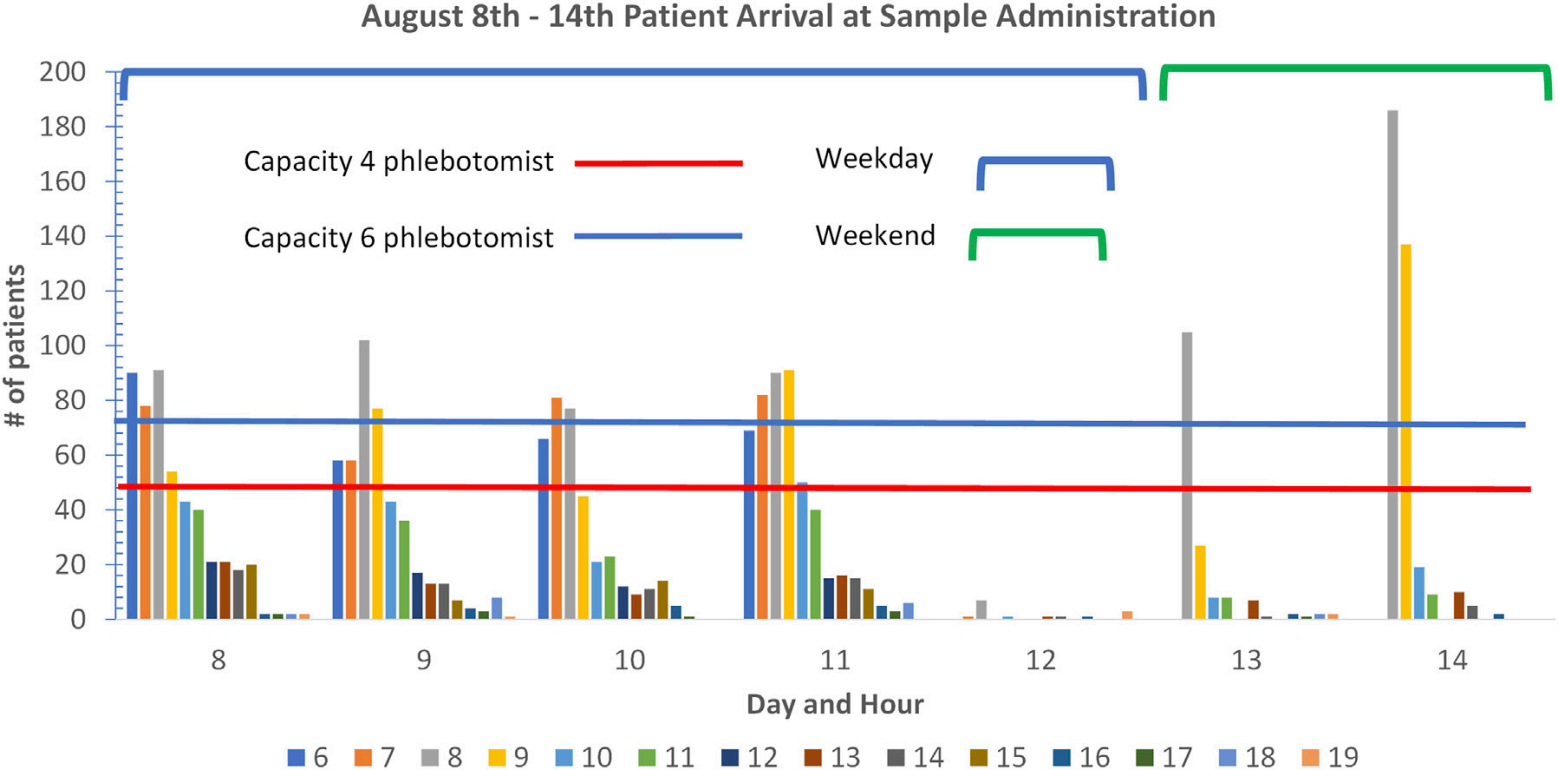
Outpatient department patient arrivals at administration in August 2022.

When looking at individual days results rather than a month, a few days showed OPD outliers, so a review was made of the patient numbers attending OPD across the 3 months July, August, and September. Each month was similar to August, shown in Figure 4; at weekends the first 2 h that the administration opens, there are many more patients than six phlebotomists can bleed.

During the weekdays in the early morning, the capacity is breached in the early hours although there is spare capacity after this time with four phlebotomists or less. There are significant numbers of wheelchair patients who take extra time as it requires phlebotomists to move away from their workstation, which can reduce overall throughput. The next stage of continuous improvement in this area is to explore the provision of more phlebotomists at peak hours or moderate the number of patients through an appointment system that can guarantee wait times equal to those shown in Figure 3.

## Discussion

Lerdsin had decided on a strategic vision of being a single touch laboratory prior to the introduction of automation, it was thought that this vision would lead to improved TAT and a reduction in human error.

In the new system, ER and IPD samples require bar-coding on arrival. They are entered into the bulk module or ES Flex from where all testing can be completed for chemistry and immunoassay with samples being centrifuged, decapped, aspirated and automatically sent for repeat testing based on the instructions for use. Samples are then recapped and sent for storage where they can be located through the Instrument Manager middleware.

The most significant impact was with the OPD patients where informatic and hardware systems were connected to streamline both patient and sample pathway. A patient only needs their national identity card to be recognized by the administration system to generate the order from the clinician directly into the LIMS as well as generate a queue number for the phlebotomy so that the patient is informed when it is their turn and which phlebotomy station to go to. The fact that patients can use their identity card to register means they do not have to bring forms with them and there is no opportunity for misinterpretation of what is requested. The patient is given a QR code that is scanned, and the tubes are pre-labelled and drawn in the right order, the sample is automatically placed in the bulk input module and processed in the same way as the IPD and ER samples. The haematology and coagulation are in-labbed on the track and are sent to a second ES Flex next to the haematology and coagulation analyzers so that samples are do not have to wait in reception and staff movement is reduced. This has simplified processes for all samples and has allowed scientific and technical staff to focus on value added activities.

The overall TAT reduction is due to the OPD process changes which can be seen by comparing Tables 3, 4 where there is a drop of around 60 min for OPD samples between May and June which is not seen in the IPD tests. The connection of the conveyor transporting OPD samples directly to the track removes them from preanalytical sample handling which then shows the reduction in TAT seen across the three departments. Primarily due to staff being able to focus on IPD and ER samples.

IPD anti-HIV tests did not show a consistent drop in TAT like the other markers which was attributed to repeat testing before a result can be released. OPD Anti-HIV tests also show a decrease in June (standalone) and stayed steady through July and August, reducing again in September and October. All values are lower than those recorded in April and May. The individual test data was reviewed and it appears that there were more results in August and September that required repeat testing before a result can be issued.

In relation to TAT, even though an increase was seen in workload volumes (see Table 5), there was a significant (*p* < 0.05) reduction in TAT with a between the mean TAT seen in April, May and June compared to July, August, September, and October. The Automation went live in July 2022 and since then there has been a decrease of 23.44%, 27.41%, and 54.24% of the TAT of IPD, ER and OPD, respectively. Individual assay TAT was also analyzed (Table 2) which showed that the aspiration of a 1-h TAT for chemistry and 2 h for immunoassay was achievable.

From the analysis of the number of patients attending the phlebotomy department (Figure 3), it could be seen that on specific days, especially at weekends, more patients would attend than could be accommodated by the six phlebotomists. When this is compared to the days when patient demand and phlebotomy capacity are matched, the patient waiting times are 10 min. This is one of the areas that will be looked at to drive continuous improvement along with the introduction of auto-verification. At Lerdsin Hospital, the innovative use of hardware, automation and informatics has already resulted in decreased TAT which improves patient care, reduction in human intervention which frees up staff to focus on quality activities and reduces the potential for error and the improved wait times in OPD has anecdotally improved patient satisfaction. The hospital management have created an advertising video interviewing patients who had used the service in the past and new patients who are praising the speed of the system.

## Summary

• Although increased laboratory turnaround time (TAT) is a risk to patient safety and increased LOS there are no agreed standards.
• Time stamps for overall TAT are difficult to measure due to sample transport or how results are sent to the clinician.
• Intralaboratory TAT has been shown to be a good indicator of quality and is measurable from the LIS.

This work represents an advance in biomedical science because it describes how the OPD process has been integrated into the automation system to allow a single touch process with TAT improvement.

## Data Availability

The original contributions presented in the study are included in the article/supplementary material, further inquiries can be directed to the corresponding author.

## Abbreviations

ER, emergency room; IMSVA, instrument manager solutions for Vitros automation; IPD, in patient; LIMS, laboratory information management system; OPD, outpatient department; TAT, turnaround time; VAS, Vitros Automation System.
